# TGF-beta receptor mediated telomerase inhibition, telomere shortening and breast cancer cell senescence

**DOI:** 10.1007/s13238-016-0322-1

**Published:** 2016-09-30

**Authors:** Lucy Cassar, Craig Nicholls, Alex R. Pinto, Ruping Chen, Lihui Wang, He Li, Jun-Ping Liu

**Affiliations:** 1 Molecular Signaling Laboratory, Department of Immunology, Central Eastern Clinical School, Monash University, Prahran, VIC 3181 Australia; 2 Institute of Aging Research, Hangzhou Normal University School of Medicine, Hangzhou, 311121 Zhejiang Province China

**Keywords:** BMPRII, TGFbeta, hTERT, telomerase, telomeres, senescence, breast cancer cells

## Abstract

Human telomerase reverse transcriptase (hTERT) plays a central role in telomere lengthening for continuous cell proliferation, but it remains unclear how extracellular cues regulate telomerase lengthening of telomeres. Here we report that the cytokine bone morphogenetic protein-7 (BMP7) induces the *hTERT* gene repression in a BMPRII receptor- and Smad3-dependent manner in human breast cancer cells. Chonic exposure of human breast cancer cells to BMP7 results in short telomeres, cell senescence and apoptosis. Mutation of the BMPRII receptor, but not TGFbRII, ACTRIIA or ACTRIIB receptor, inhibits BMP7-induced repression of the *hTERT* gene promoter activity, leading to increased telomerase activity, lengthened telomeres and continued cell proliferation. Expression of hTERT prevents BMP7-induced breast cancer cell senescence and apoptosis. Thus, our data suggest that BMP7 induces breast cancer cell aging by a mechanism involving BMPRII receptor- and Smad3-mediated repression of the *hTERT* gene.

**Electronic supplementary material:**

The online version of this article (doi:10.1007/s13238-016-0322-1) contains supplementary material, which is available to authorized users.

## INTRODUCTION

Human telomerase reverse transcriptase (hTERT) is the catalytic subunit of telomerase required for telomere maintenance and continuous cell proliferation in most cancers (de Lange, 2002; Blasco, 2005; Blackburn et al., 2006; Shay and Wright, 2006). The availability of hTERT, however, is under a strict regulation at the gene transcription level (Kyo and Inoue, 2002; Takakura et al., 2005). A number of cellular factors have been implicated as playing significant roles in regulating hTERT gene expression, among which are the proto-oncogene Sp1/Sp3, c-myc, Ets2 transcription factor (Wu et al., 1999; Kyo et al., 2000; Xu et al., 2008; Horn et al., 2013; Huang et al., 2013; Borah et al., 2015; Cheng et al., 2015), and the Smad3 repressor (Hu et al., 2006; Li et al., 2006). Since the hTERT gene is repressed during cell differentiation (Bestilny et al., 1996; Holt et al., 1996; Parsch et al., 2004; Li et al., 2005), extracellular factors that regulate cell differentiation may be involved in programming hTERT gene expression and thereby telomerase-mediated maintenance of telomere homeostasis (Li et al., 2005, 2006; Li and Liu, 2007). We and others show that TGF-β is involved in cancer cell proliferation under certain conditions (James et al., 2005; Li et al., 2005, 2006; Biswas et al., 2007; Galliher and Schiemann, 2007), suggesting that extracellular cytokines regulate the hTERT gene.

Bone morphogenetic proteins (BMPs) operate though autocrine and paracrine mechanisms to regulate cell proliferation, differentiation and apoptosis during development (Patel and Dressler, 2005; Varga and Wrana, 2005). BMPs act on their cell membrane type I and II receptors of serine/theonine kinases (Hogan, 1996; Shi and Massague, 2003; Varga and Wrana, 2005) and induce phosphorylation and nuclear signaling of Smad1, Smad5 and Smad8 proteins (Attisano and Wrana, 2002; Massague et al., 2005; Varga and Wrana, 2005). Bone morphogenetic protein-7 (BMP7; osteogenic protein-1) is a potent inducer of cell differentiation required for vertebrate development. However, the function of BMP7 in adult tissues remains to be fully explored (Hogan, 1996; Wang and Hirschberg, 2004a; Patel and Dressler, 2005; Buijs et al., 2007b; Sugimoto et al., 2007; Zeisberg et al., 2007). BMP7 maintains the phenotypes of epithelium and endothelium against mesenchymal transition (Buijs et al., 2007b; Zeisberg et al., 2007). BMP7 is also readily detectable in solid tumors including breast and prostate carcinomas, and involved in regulating tumor cell proliferation and transition (Miyazaki et al., 2004; Yang et al., 2005; Alarmo et al., 2006, 2007; Buijs et al., 2007b; Notting et al., 2007; Rothhammer et al., 2007; Zeisberg et al., 2007). Previous studies show that BMP7 induces Smad5 signaling to antagonize TGF-β-induced Smad3 nuclear accumulation and block the transcriptional up-regulation of the Smad3 target CAGA box promoter activity in mesangial cells (Wang and Hirschberg, 2004b), but in breast cancer cells, BMP7 appears inhibiting TGF-β-induced Smad3 target CAGA box promoter activity (Buijs et al., 2007a).

In the present study, we found that telomerase activity in human breast cancer cells is inhibited by BMPRII receptor-mediated signaling. Treatments of the cells in cultures with BMP7 in every two-day for two weeks lead to telomere shortening and cell senescence. Forced gene expression of hTERT inhibits BMP7-induced breast cancer cell senescence and death. In addition to stimulating Smad1, Smad5 and Smad8 phosphorylation and nuclear translocation, BMP7 also induces Smad3 phosphorylation and nuclear accumulation in breast cancer cells and silencing Smad3 abrogates BMP7-induced telomerase inhibition. Thus, we show that BMP7 inhibits telomere maintenance by a mechanism involving BMPRII receptor and Smad3 signaling to suppress hTERT gene expression in breast cancer cells.

## RESULTS

### BMP7 induces telomerase inhibition and telomere shortening in cultured breast cancer cells

**Figure 1:**
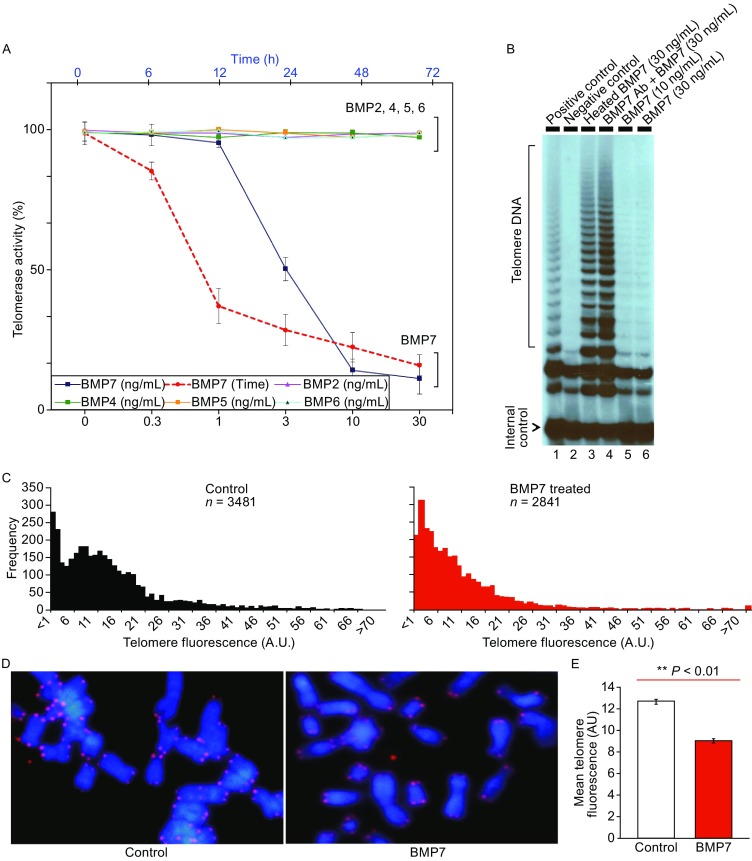
**BMP7 induces inhibition of telomerase activity and shortening of telomeres in cultured breast cancer cells**. (A) BMP7 induces a concentration- and time-dependent inhibition of telomerase activity in cultured human breast cancer MCF-7 cells. Cells were incubated with different concentrations of BMPs as indicated for 48 h (solid lines), or with BMP7 (10 ng/mL) for different periods of time as indicated (dot line). Telomerase activity was determined by measuring newly synthesized telomeric DNA and quantitated by densitometry. The results are presented in mean ± SD from thee determinations. (B) Effects of BMP7 antibodies or heating denatured BMP7 on telomerase activity. BMP7 with or without heating at 80 °C for 2 min was incubated in the presence or absence of BMP7 antibodies (1:100 dilution) in cultured cells. Positive control is 1 in 10 dilution of MCF7 cell nuclear telomerase extracts and negative control is the Tris buffer used for telomerase extraction. Telomere DNA produced and internal control are indicated. (C) Effect of BMP7 on telomere length in MCF-7 cells. Cells were treated with or without BMP7 (30 ng/mL) for 15 h followed by replacement with fresh medium on every second day for two weeks. Telomeres were determined by Q-FISH and the distributions of telomere length are presented by fluorescence intensity versus frequencies. (D) Illustrative micrographs of fluorescence-labeled telomeres in metaphase spreads. (E) Histograms of mean telomere fluorescence intensities (± S.E.M.) in control and BMP7-treated cells

To investigate a potential involvement of the BMP family in telomerase activity, we examined the effects of BMP2, BMP4, BMP5, BMP6 and BMP7 on telomerase activity by spiking the medium of cultured human breast cancer MCF-7 cells with purified recombinant human cytokines. Incubation of MCF-7 cells with different concentrations of BMP7 for 48 h resulted in more than 70% inhibition of telomerase activity (Fig. 1A). The median inhibitory concentration (IC50) of BMP7 was 4 ± 0.5 ng/mL and maximal inhibitory concentration was 20 ± 0.8 ng/mL (*n* = 3) (Fig. 1A), concentrations that are within the levels of BMPs administered and observed in vivo (Simic et al., 2006; Ma et al., 2007).

In contrast to BMP7, incubations of the cells with BMP2, BMP4, BMP5 or BMP6 showed no significant inhibitory effect on the telomerase activity (Fig. 1A). Consistent with a specific effect, BMP7 antibodies abrogated the inhibition, and denatured BMP7 had no effect (Fig. 1B). BMP7 also elicited telomerase inhibition in a time-dependent manner (Fig. 1A), with the inhibition occurring in about 24 h and lasted for 2–3 days. In addition to inhibiting telomerase activity, BMP7 consistently induced a significant reduction in the size of telomeres. Pulsatile treatments of cultured MCF-7 cells with BMP7 (30 ng/mL) for 15 h thee times a week over two weeks resulted in significantly shortened telomeres (Fig. 1C and 1D), as revealed by quantitative fluorescence in situ hybridization (Q-FISH) using a specific telomeric DNA probe in the metaphase cell spread preparations. As shown in Fig. 1C, BMP7 treatments induced a marked shift of the peak of the telomere fluorescence intensity signals between the control and BMP7-treated groups. While the majority of the telomere sizes were distributed with high frequency at the 6–26 fluorescence units (peaking at 11) in the control cells, the peak of telomere signals was at the 6th fluorescence unit in the BMP7-treated group exhibiting much shorter telomeres than that in the control group. Comparison of the two telomere fluorescence distribution peaks showed *P* value to be less than 0.0001, by Kolmogorov-Smirnov test. On average, the telomeres in the BMP7-treated group were 25%–30% shorter than the telomeres in the normal control cells (Fig. 1D and 1E). Thus, the data together clearly showed that BMP7 induced inhibition of telomerase activity and shortening of telomeres in cultured human breast cancer cells.

### BMP7 induces breast cancer cell senescence and death by a mechanism dependent on telomerase inhibition

**Figure 2:**
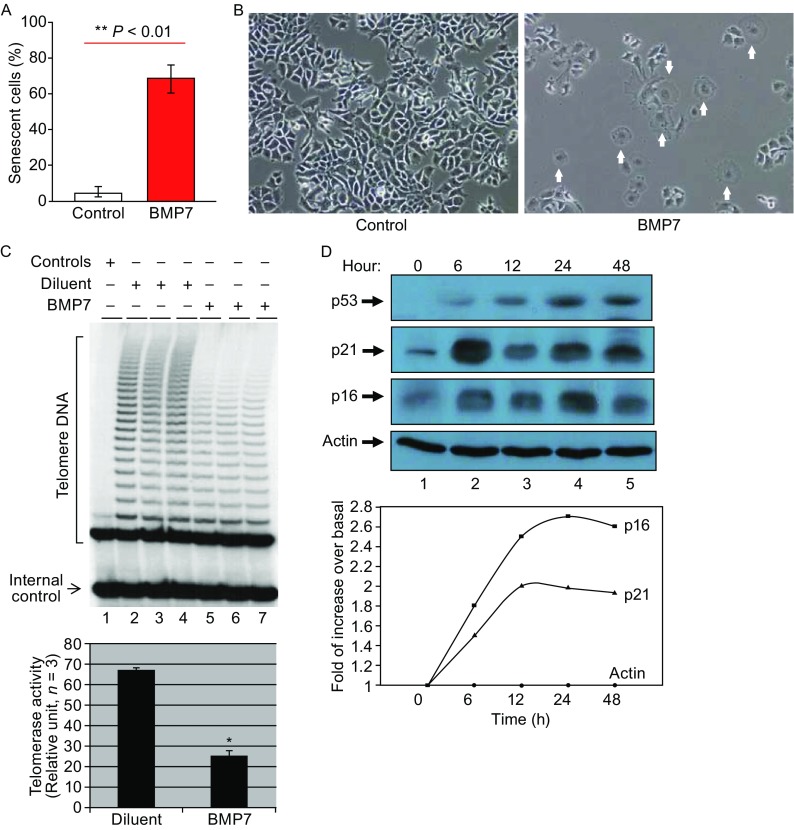
**BMP7 induces cancer cell senescence**. (A) BMP7 induces an increase in cancer cell senescence. MCF-7 cells were incubated with or without BMP7 (30 ng/mL) for 15 h three times a week for two weeks. Senescence-like cells were counted in multiple micrographs and presented as means with comparison between the control and BMP7-treated groups. The results are in mean ± SD from five determinations. (B) Representative micrographs of cultured MCF-7 cells photographed under phase contrast microscope at 10X magnification. Senescence-like cells are indicated by arrows. (C) Inhibition of telomerase activity in MCF-7 cell cultures treated with BMP7 for 15 h three times a week for two weeks. The result was from one of two assays. (D) Increases in p16, p53 and p21 in the MCF-7 cells treated with BMP7 (10 ng/mL) for different periods of time, determined by Western blotting. The data presented in top panel were from one of two similar experiments, and the data in bottom panel are means of two determinations

With the possible mechanisms of BMP7 action on the cell surface to regulate gene expression programs and cellular phenotypes, we treated cultured breast cancer cells with BMP7 overnight with repeats in every two-day for two weeks and examined cell senescence and death. In the BMP7 treated cell cultures, we observed the cells characteristics of enlarged and flattened cell morphology, greater cytoplasm/nuclear ratio, and expressions of cell senescence markers such as β-galactosidase and p16 (Janzen et al., 2006; Molofsky et al., 2006). As shown in Fig. 2A, treatment of MCF-7 cells with BMP7 (30 ng/mL, 15 h in every two-day for two weeks) resulted in a marked increase in the incidence of cell senescence (Fig. 2A). The increase in cell senescence in the BMP7-treated cultures was associated with reduced cell numbers (Fig. 2B) and protein concentrations (not shown), decreased telomerase activity (Fig. 2C). The inhibition of telomerase activity in these cells was by 60%–70%. Consistent with cell senescence, BMP7-treated cell cultures showed increased p53, p21 and p16 (Fig. 2D). The levels of p16, p53 and p21 were 2–5 folds of controls plateaued in 24 h of BMP7 treatment (Fig. 2D). Thus, our data showed that prolonged exposure to BMP7 induced tumor cell growth arrest, senescence and death.

**Figure 3:**
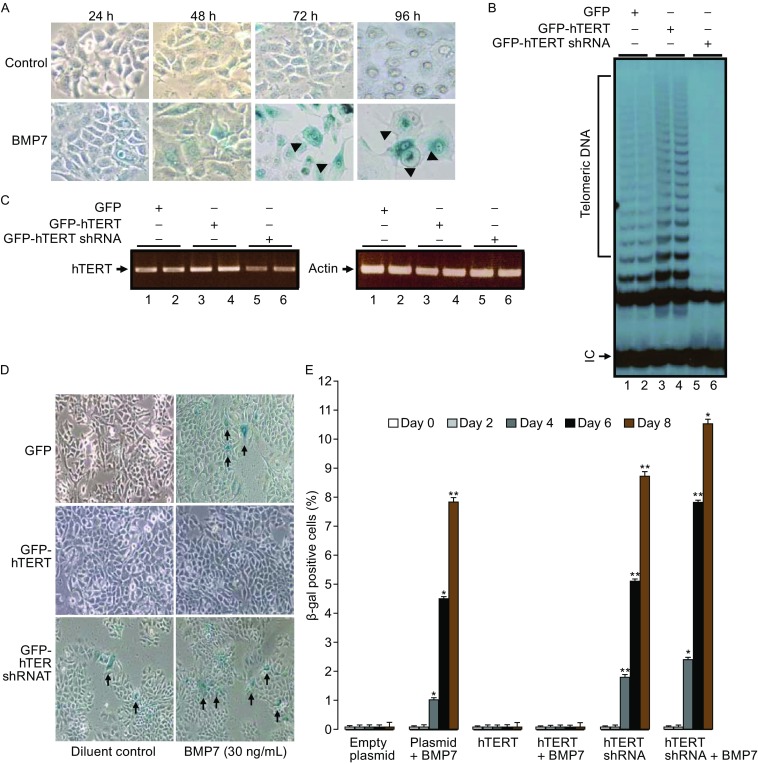
**BMP7-induced breast cancer cell senescence is dependent on hTERT gene repression**. (A) β-galactosidase activity determined by β-Gal staining in cultured MCF-7 cells treated with BMP7 (10 ng/mL) for different periods of time. Results were from phase contrast microscopy at 20X magnification. Arrowed are enlarged β-galactosidase positive senescent cells. (B) Up- and down-regulation of telomerase activity by GFP-hTERT and GFP-hTERT shRNA. MCF-7 cells were transfected for 24 h. Total cell lysates were subjected to telomerase activity assay. De novo synthesized telomeric DNA reflecting telomerase activity and internal loading control are indicated. (C) Over- and under-expression of hTERT by gene transfection. MCF-7 cells transfected with GFP, GFP-hTERT or GFP-hTERT shRNA were extracted for mRNA. RT-PCR was performed to verify hTERT gene expression with actin as control. (D) Effects of hTERT over- and under-expression on BMP7-induced breast cancer cell senescence. MCF-7 cells expressing basal, increased or decreased levels of hTERT were examined for cell senescence by β-Gal staining. Positive cells with blue staining are indicated by arrows. (E) Quantification of β-Gal positive cells in time course studies. Results are mean plus standard errors (*n* = 3) Asterisk denotes p value less than 0.001 when compared with controls

To further determine BMP7-induced breast cancer cell senescence and the role of telomerase inhibition, β-Gal staining was carried for the β-galactosidase activity in MCF-7 cells treated with BMP7 for different periods of time. As shown in Fig. 3A, β-Gal positivity was observed in the enlarged cells (arrowed) in MCF-7 cell cultures that were treated with BMP7 in 72 h or 96 h, confirming that BMP7 treatment is associated with breast cancer cell senescence. To investigate if telomerase inhibition induced by BMP7 mediated BMP7-induced cancer cell senescence, we carried out over- and under-expression of hTERT with GFP-hTERT and GFP-hTERT shRNA gene expression systems respectively, using GFP alone as control. In 24 h of transfection, transfected cells were sorted to purify the different transformants by fluorescence activated cell sorter (FACS). Telomerase activity (Fig. 3B) and hTERT mRNA (Fig. 3C) was determined to verify the changes of different levels of telomerase and hTERT gene expression by TRAP and RT-PCR respectively. Significantly, treatment of the GFP, GFP-hTERT and GFP-hTERT shRNA transfected cells with or without BMP7 resulted in different patterns of β-Gal staining. As shown in Fig. 3D and 3E, BMP7 (30 ng/mL) induced cell senescence in GFP transfected cells, and similarly, hTERT shRNA also induced cell senescence without BMP7 treatment. However, expression of recombinant hTERT prevented BMP7-induced senescence and hTERT shRNA increased BMP7-induced senescence (Fig. 3D and 3E). Comparison of BMP7 alone or hTERT shRNA alone with BMP7 plus hTERT shRNA showed a significant difference between BMP7 alone and BMP7 plus hTERT shRNA (7.7 ± 0.55 versus 10.5 ± 0.82, *P* < 0.01), or between hTERT shRNA alone and hTERT shRNA plus BMP7 (8.7 ± 0.62 versus 10.5 ± 0.82, *P* < 0.03). These findings that BMP7 plus hTERT shRNA induced cell senescence to a greater degree than that by BMP7 or hTERT shRNA alone suggest that hTERT suppression is involved but not the only factor in mediating BMP7-induced cell senescence.

**Figure 4:**
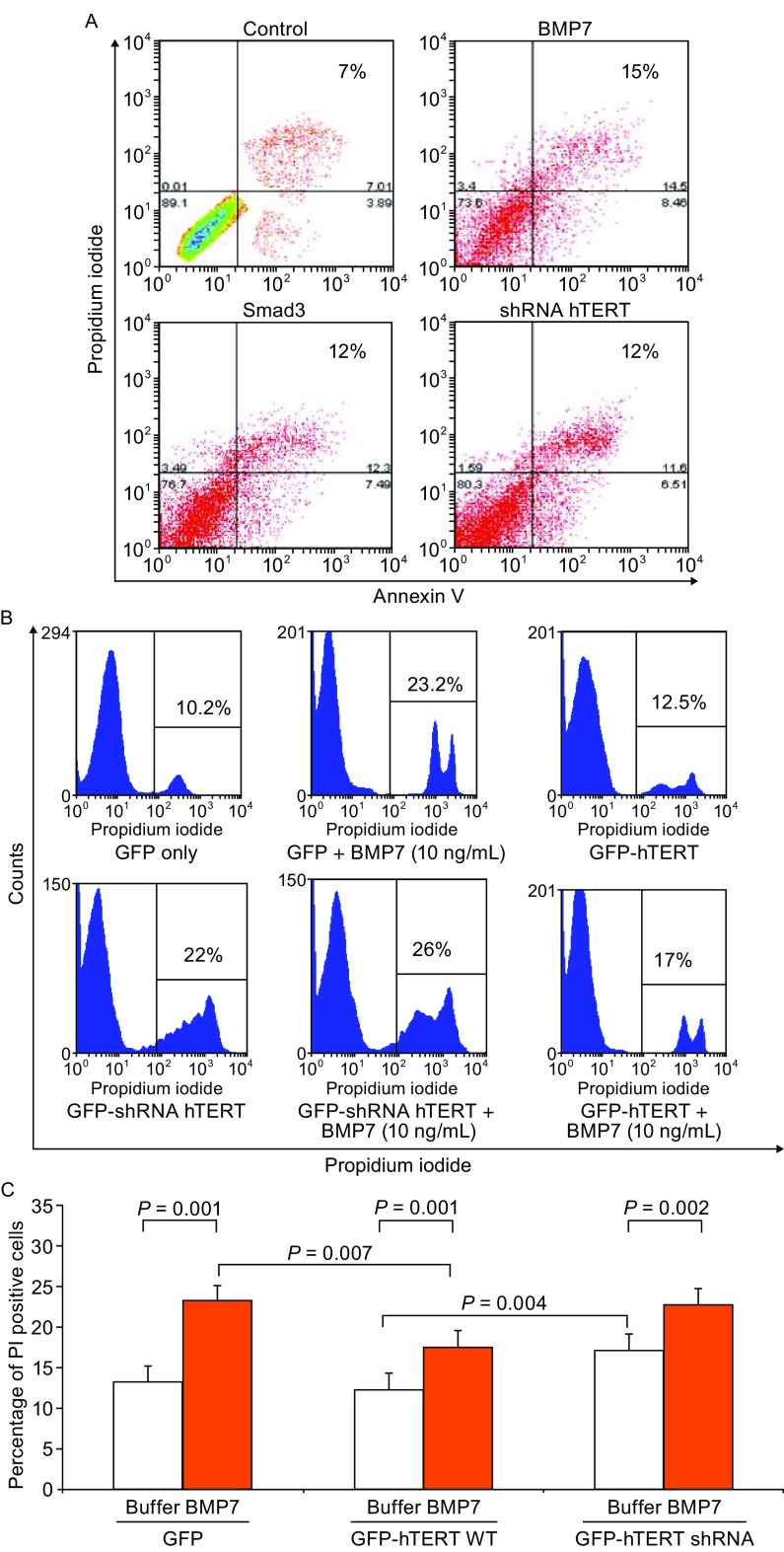
**BMP7 induces hTERT-dependent breast cancer cell apoptosis**. (A) BMP7 induces breast cancer cell apoptosis. MCF-7 cells were treated with BMP7 (10 ng/mL), or transfected with Smad3 or hTERT shRNA expression plasmids for 48 h. Cells were stained with Annexin V and propidium iodide (PI), and analyzed by FACS. (B) Effects of over- and under-expression of hTERT on BMP7-induced breast cancer MCF-7 cell death. Cells were transfected with pEGFP, pEGFP-hTERT or pEGFP-hTERT shRNA for 24 h, followed by FACS sorting. GFP-positive cells were cultured for 15 h before being treated with BMP7 (10 ng/mL, 24 h) and analysed by apoptotic cell staining in FACS. (C) Comparison and statistical analysis of the mean inhibitory effect of recombinant hTERT on BMP7-induced cell death. The data are mean ± SD from eight similar experiments. P values were from student t-tests

To explore BMP7 instigation of breast cancer cell apoptotic cell death, we stained the BMP7 treated cells with annexin V and propidium iodide (PI), and analyzed double positive cells in FACS. Incubation of MCF-7 cells with BMP7 (30 ng/mL) for 24 h resulted in a significant increase in the percentage of annexin V- and PI-positive cells (Fig. 4A). The number of apoptotic cells in 24 h of BMP7-treated cell cultures was doubled when compared to the basal levels in control group. To examine the involvement of telomerase inhibition in breast cancer cell apoptosis, we observed an increased response of apoptosis to the transient overexpression of Smad3 that is an hTERT gene repressor or to the transient expression of the hTERT shRNA to silence the hTERT gene. The levels of induced apoptosis by BMP7 treatment, Smad3 overexpression or hTERT shRNA were within 10%–20% of total cells (Fig. 4A), consistent with a specifically increased incidence of apoptosis due to telomerase inhibition.

To further attest the role of hTERT in the BMP7-induced cell death, we similarly transfected MCF-7 cells with recombinant GFP-hTERT, GFP-hTERT shRNA or GFP only gene expression plasmids, isolated the GFP-positive transformants by FACS, and treated the cells with BMP7 (10 ng/mL) for 24 h (Fig. 4B and 4C). In the homogenously transfected cell cultures, we found that BMP7 treatment doubled the number of apoptotic cells of control, and hTERT shRNA induced a significant cell death in parallel (Fig. 4B). In addition, hTERT shRNA did not potentiate the levels of cell death induced by BMP7 (Fig. 4B), suggesting a possibly shared mechanism. Overexpression of hTERT wild type significantly reduced the levels of BMP7-induced cell death (Fig. 4B). In average, the gene expression GFP-hTERT reduced the levels of cell death induced by BMP7 from 19% to 14% (*P* = 0.007) (Fig. 4C). These data demonstrated that hTERT repression mediated the breast cancer cell apoptosis induced by BMP7 at least in part. Furthermore, we also noted that hTERT did not completely prevent BMP7-induced cells. As shown in Fig. 4C, although hTERT overexpression reduced BMP7-induced cell death significantly, BMP7 treatment of GFP-hTERT transformants still showed more cell death than the GFP-hTERT transformants without BMP7 treatment (*P* = 0.001) (Fig. 4C). Consistently, while hTERT shRNA induced a significant increase in apoptosis (*P* = 0.004), the cell death induced by the combination of hTERT shRNA and BMP7 was greater than hTERT shRNA alone (*P* = 0.002) (Fig. 4C). Thus, over and above hTERT repression, other mechanism(s) may exist to participate in mediating BMP7-induced breast cancer cell senescence as well as apoptosis.

### BMP7 repression of the hTERT gene is mediated by Smad3 signaling

**Figure 5:**
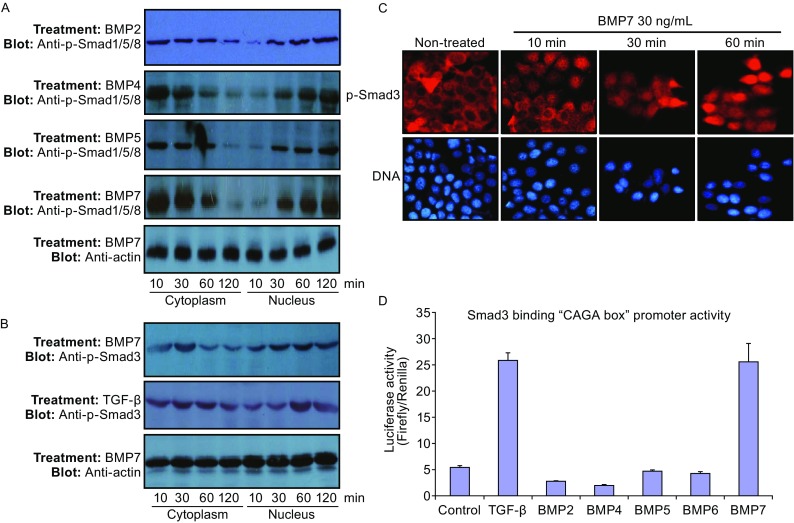
**BMP7 induces Smad3 phosphorylation, nuclear translocation and gene transcriptional activity**. (A) Smad1/5/8 phosphorylation and nuclear accumulation stimulated by BMP7, BMP2, BMP4 and BMP5. MCF-7 cells were incubated with the cytokines (30 ng/mL) for different time periods as indicated. Cells were fractionated by differential centrifugation for the cytoplasmic and nuclear fractions. Phosphorylation of Smad1/5/8 complex in response to the different cytokines as indicated was examined by Western blotting using specific anti-phosphorylated Smad1/5/8 antibodies. (B) Smad3 phosphorylation and nuclear accumulation stimulated by BMP7 and TGF-β. MCF-7 cells were incubated with BMP7 or TGF-β (30 ng/mL) for 10, 30, 60 and 120 min. Cells were fractionated and Smad3 phosphorylation was determined Western blotting using specific anti-phosphorylated Smad3 antibody. Protein loading controls in each fraction were monitored by Western blotting using anti-actin antibodies. Data were representatives of at least thee similar experiments. (C) Immunofluorescence staining of phosphor-Smad3 in BMP7-treated MCF-7 cells at different time points. Cells were treated with BMP7 for different periods of time as indicated. Micrographs of immunofluorescence staining using specific antibody against phosphor-Smad3 and staining for the nuclei are shown. (D) BMP7 activation of Smad3 inducible (CAGA)12 Luc promoter. Cells transfected with pCAGA12 Luc were treated with different cytokines as indicated at the concentrations of 10 ng/mL for 24 h. Luciferase activity was determined as described in MATERIALS AND METHODS. Data are mean ± SD from four determinations

To investigate the mechanisms of the inhibitory effect of BMP7 on telomerase activity, we determined hTERT gene expression as a possible function of the activation of Smad proteins. BMP7 stimulated the phosphorylation and nuclear retention of Smad1/5/8 complex, similar to that by BMP2, BMP4 and BMP5 (Fig. 5A). In a preliminary experiment, we observed no apparent effect of Smad1, Smad5 and Smad8 on telomerase inhibition (not shown). However, we found surprisingly that Smad3 responded to BMP7. As shown in Fig. 5B, incubation of cultured MCF-7 cells with BMP7 for different periods of time from 10 min to 2 h resulted in Smad3 phosphorylation. The phosphorylation was gradually increased and the maximal levels of phosphorylation were two-thee folds of that under basal conditions in a time-dependent manner (Fig. 5B). In addition, the phosphorylation of Smad3 occurred in association with the phosphorylated protein nuclear accumulation from ten min of BMP7 treatment, which lasted for two h of observation (Fig. 5B). As controls, Smad3 phosphorylation was also induced by TGF-β stimulation (Fig. 5B), but no Smad3 phosphorylation was observed in the cells treated with BMP2, BMP4 or BMP5 (not shown). Immunofluorescence staining of phosphor-Smad3 intracellular distribution confirmed the data obtained by cellular fractionation. As shown in Fig. 5C, predominant cytoplasmic staining of phospho-Smad3 was observed in non-treated MCF-7 cells, whereas predominant nuclear staining of phosphor-Smad3 was observed in some cells 10 min after BMP7 treatment and in most cells 30 or 60 min after the BMP7 treatment.

To further determine the increased Smad3 activity in response to BMP7 stimulation, we measured Smad3 gene transcriptional activity using a Smad3 responsive luciferase gene reporter assay in MCF-7 cells treated with or without BMP7. MCF-7 cells were transfected with Smad3-responsive gene promoter (pGL3-(CAGA)_12_-Luc luciferase reporter (Dennler et al., 1998)) for 24 h, and the transfected cells were further treated with 30 ng/mL of BMP7, TGF-β, BMP2, BMP4, BMP5 or BMP6 for 48 h. As shown in Fig. 5D, BMP7 or TGF-β induced marked increases in the luciferase reporter gene activity that is under the transcriptional control of the Smad3-specific promoter. The increased luciferase activity induced by BMP7 was four-five folds of control, which was comparable to that induced by TGF-β (Fig. 5D). In contrast, BMP2, BMP4, BMP5 or BMP6 did not elicit any significant increase in the luciferase activity driven by Smad3 responsive promoter (Fig. 5D). These data further confirmed that BMP7 triggered the nuclear signaling of Smad3 in breast cancer cells.

**Figure 6:**
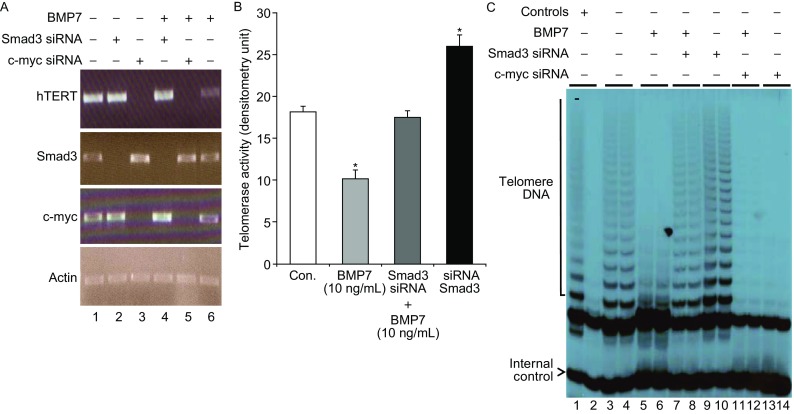
**BMP7 induced inhibition of the hTERT gene expression and telomerase activity requires Smad3**. (A) Effects of silencing Smad3 and c-myc on BMP7-induced repression of hTERT mRNA. MCF-7 cells were transfected with (lanes 2–6) or without (lane 1) different customer synthesized small interference RNA (siRNA) as indicated for 48 h. Cells were then treated with (lanes 4–6) or without (lanes 1–3) BMP7 (10 ng/mL) for 24 h. Gene expressions of hTERT, Smad3, c-myc and actin were determined by measuring their respective mRNA by RT-PCR using specific primers. (B) Quantification of telomerase activity from breast cancer cells treated with BMP7 plus or minus the gene silencing of Smad3. Telomerase activity was measured by TRAP and the levels of telomerase activity were quantitated by scanning of the autoradiographs with densitometry. Data are mean plus standard errors (*n* = 3) and asterisk notes *P* < 0.001 compared with non-treated control. (C) Effects of silencing Smad3 and c-myc on BMP7-induced inhibition of telomerase activity. MCF-7 cells were transfected with siRNA specific to Smad3 (lanes 7–10) or c-myc mRNA (lanes 11–14) for 48 h. Cells were then treated with BMP7 (10 ng/mL) (lanes 5–8, 11–12) or diluent (lanes 1–4, 9–10, 13–14) for 24 h. Lanes 1 and 2 were controls: lane 1 was a positive control of telomerase extracts and lane 2 was a negative control treated with RNase A. These data presented were representative of one of at least three similar experiments

With the observed correlative changes of Smad3 nuclear signaling (Fig. 5B, 5C and 5D) and telomerase inhibition induced by BMP7 (Fig. 1A), we next investigated a causal role of the BMP7-induced Smad3 signaling in mediating the BMP7-induced hTERT gene repression by silencing the Smad3 gene and then determining if the inhibitory effect of BMP7 on the hTERT gene expression is mitigated. As shown in Fig. 6, silencing Smad3 gene expression resulted in increased gene expressions of c-myc and hTERT (Fig. 6A, *lane 2* or *4*, *versus lane 1*), and increased telomerase activity (Fig. 6B, *lane 9*–*10 versus lane 3*–*4*). In contrast, silencing c-myc gene expression resulted in inhibition of the hTERT gene expression (Fig. 6A, *lane 3 or 5, versus lane 1*), or telomerase activity (Fig. 6B, *lane 11*–*14 versus lane 3*–*4*), consistent with previous findings that Smad3 is a repressor and c-myc is a transcription factor of the hTERT gene respectively (Wu et al., 1999; Kyo and Inoue, 2002; Takakura et al., 2005; Li et al., 2006). However, silencing Smad3 markedly relaxed the BMP7 inhibition of the hTERT gene expression and telomerase activity (*lane 4 versus lane 6* of Fig. 6A, and *lanes 7*–*8 versus lanes 5*–*6* of Fig. 6B). These findings that knocking down Smad3 gene expression disrupted BMP7-induced telomerase inhibition clearly suggested that Smad3 was required in BMP7-induced telomerase inhibition in human breast cancer cells. Thus, BMP7 employed Smad3 to repress the hTERT gene, inhibit telomerase activity and induce telomere shortening in cultured breast cancer cells.

### Roles of TGF-β family cytokine receptors in regulating the hTERT gene, telomerase activity and telomere length

**Figure 7:**
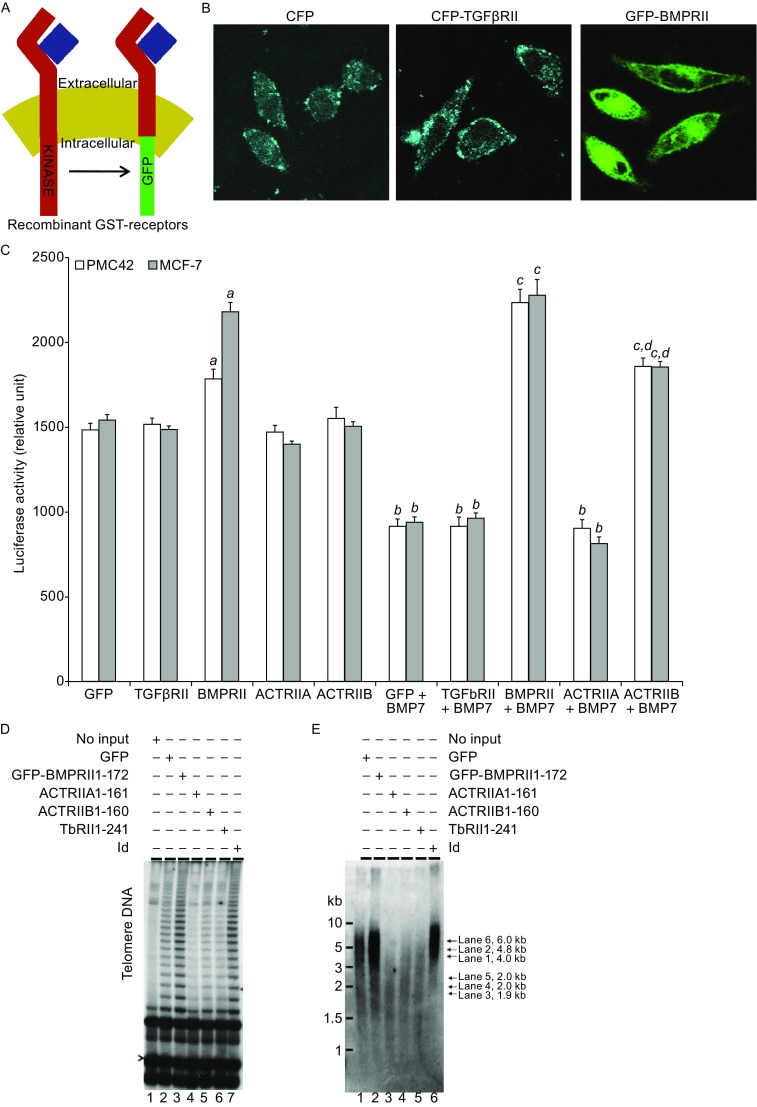
**Truncation of BMPRII receptor blocks BMP7-induced repression of the hTERT gene and induces increased telomerase activity and telomere length in breast cancer cells**. (A) Schematic presentation of wild type TGF-β receptor and recombinant truncated GFP fusion receptors. (B) Expression of truncated TGF-β or BMP type II receptors in MCF-7 breast cancer cells. Cells were transiently transfected with CFP control, CFP-BMPRII 1–172 or GFP-TGFβRII 1–214 for 24 h before the cells were fixed and observed by confocal microscopy. (C) Effects of mutations of TGF-β family receptors on the hTERT gene promoter activity. MCF-7 (hatched bars) and PMC42 (white bars) breast cancer cells were co-transfected for 24 h with hTERT gene promoter p330 upstream of a luciferase reporter gene together with GFP, GFP-TGF-β RII 1–214, GFP BMPRII 1–172, GFP ACTRIIA 1–161 or GFPACTRIIB 1–160 for 24 h. Cells were treated with or without BMP7 (10 ng/mL, 4 h) followed by luciferase activity assay as described in Materials and Methods. Data are mean ± SD from three experiments determined in triplicate. a, *P* < 0.01 compared with GFP only control; b, *P* < 0.01 compared with corresponding gene transfection without BMP7 treatment; c, *P* < 0.01 compared with GFP control treated with BMP7; and d, *P* < 0.01 compared with BMPRII mutation treated with BMP7. (D) Effects of truncated TGF-β family receptors on telomerase activity. MCF-7 cells were stably transfected with various gene expression plasmids as indicated and the transfected cells were selected in cultures for approximately 70 days. GFP-positive cells sorted by FACS were examined for telomerase activity as described in Materials and Methods. Telomeric DNA and internal control are indicated by arrows. (E) Effects of truncated TGF-β family receptors on telomere length. Cells with stable expression of TGF-β family cytokine receptor truncation mutants for ~70 days in cultures were analyzed for telomere length as described MATERIALS AND METHODS. The average sizes of telomere repeat fragment (TRF) under different gene expression conditions are indicated on the right of the autoradiograph with lane numbers. Data were representatives of multiple measurements from 2–3 experiments

BMP7 has been shown to bind BMPRII or ACTRIIB receptors to induce intracellular signaling (Shi and Massague, 2003). To establish a specifically receptor-mediated effect of BMP7 on telomerase inhibition, we performed dominant negative gene expression by truncating the cytoplasmic domains of all four TGF-β family cytokine receptors, BMPRII, TGFβRII, ACTRIIA and ACTRIIB. We determined the effects of forced expression of these receptor truncation mutants on hTERT gene promoter activity, telomerase activity and telomere length. Recombinant TGFβRII, BMPRII (Fig. 7A), ACTRIIA and ACTRIIB (not shown) receptors with missing intracellular domains were all expressed on the MCF-7 breast cancer cell surface as expected. Examination of their effects on hTERT gene promoter activity following transient gene expressions of these receptor mutants showed distinct patterns. Mutation of BMPRII receptor resulted in a significant elevation (20%–30%, *P* < 0.01) of the basal hTERT gene promoter activity compared with the controls expressing GFP only in MCF-7 breast cancer cells (Fig. 7B), suggesting that BMPRII receptor mediates an endogenous inhibitory signal to the repression of the hTERT gene promoter activity in breast cancer cells. Treatment of the different receptor mutants with BMP7 resulted in a consistent repression of the hTERT gene promoter activity compared with BMP7-untreated controls (~40%, *P* < 0.01) (Fig. 7B). Mutation of BMPRII receptor blocked the inhibitory effect of BMP7, whereas mutation of TGFβRII or ACTRIIA receptors did not affect BMP7 inhibition of the hTERT gene promoter (Fig. 7B), suggesting that BMPRII receptor, but not TGFβRII or ACTRIIA receptors, mediates the inhibition of the hTERT gene induced by BMP7. In addition, mutation of ACTRIIB receptor also inhibited BMP7-induced repression of the hTERT gene promoter activity albeit to a significantly less extent than that induced by BMPRII receptor mutation (Fig. 7B), suggesting that ACTRII receptor plays a less dominant role than that played by BMPRII receptor in mediating BMP7-induced hTERT gene repression.

To investigate the effects of TGF-β family cytokine receptor mutations on endogenous telomerase activity and telomere length in breast cancer cells, we established stable gene expressions of the receptor mutants along with negative and positive controls by repeated antibiotic selection and FACS sorting for GFP-positive cells. Stable expression of the truncated TGF-β family cytokine RII receptors for ~2.5 months resulted in a significantly increased telomerase activity (Fig. 7C), comparable to a positive control induced by overexpression of the Ets Id (Xu et al., 2008). In contrast, expressions of TGF-βRII, ACTRIIA or ACTRIIB all resulted in decreased telomerase activity compared with GFP only control (Fig. 7C). Measurement of telomere repeat fragments (TRF) in the breast cancer cell lines stably expression TGF-β family cytokine receptor mutants showed consistent results. As shown in Fig. 7D, cells with dominant negative BMPRII exhibited lengthened TRF compared with that in cells expressing GFP only (4.8 kb vs. 4.0 kb). Conversely, cells expressing dominant negative TGFβRII or AVTRII receptors showed decreased TRFs (Fig. 7D). These stimulatory effects of BMPRII and ACTRIIB mutation were consistent with the results obtained from transient gene expression experiments in the absence of BMP7 suggesting that wild type BMPRII as well as ACTRIIB receptors were involved in mediating a negative regulation of telomerase activity. In contrast, the inhibitory effects of TGFbRII and ACTRIIA mutations were only observed in the cells stably expressing the receptor mutants, suggesting a possibly involvement of other unidentified mechanisms that require further investigation.

To investigate whether or not the observed telomerase inhibition induced by BMP7, mediated by BMPRII receptor, is limited to the MCF-7 breast cancer cell line, we exploited another breast cancer cell line—PMC42 cells. Treatment of PMC42 cells with BMP7 resulted in a significant repression of the hTERT gene promoter. The extent of BMP7-induced hTERT gene promoter inhibition was similar to that induced by BMP7 in MCF-7 cells (Fig. 7B). Overexpression of BMP RII receptor similarly blocked BMP7-induced hTERT gene repression in PMC42 breast cancer cells (Fig. 7B). These data suggested that BMP7 negatively regulated telomerase activity though the BMPRII receptor in more than one breast cancer cell line. Together, these experiments demonstrated that BMPRII and to a less degree ACTRIIB receptors mediated BMP7-induced hTERT gene repression, inhibition of telomerase activity and shortening of telomeres in breast cancer cells.

## DISCUSSION

Consistent with the notion that telomerase is subject to membrane receptor-mediated extracellular micro-environmental regulation (Ogawa et al., 2006; DamLe et al., 2012; Jacob et al., 2016), in this study, we demonstrate that BMPRII receptor is involved in mediating recombinant human BMP7 inhibition of telomerase activity and telomere maintenance in cultured human breast cancer cells, resulting in cancer cell senescence and death. Our data demonstrate that telomerase maintenance of telomeres in tumor cells is subject to regulation by extracellular cytokine and that the BMP7 pathway plays a significant role in the negative regulation of telomerase activity and telomere maintenance in cancer. The maintenance of telomeres by telomerase in cancer is therefore likely to be reprogrammable by a defined extracellular environment, probably by a combination of extracellular factors with BMP7 to be an inhibitory cytokine of telomerase-mediated telomere homeostasis.

The BMP7-induced occurrence of breast cancer cell senescence appears several days after treatment, following the inhibition of telomerase activity. Pulsatile treatments of breast cancer cells with BMP7 for two weeks are effective to induce a significant shortening of telomeres, marked increase in cell senescence and drastic loss of cultured cells. Consistently, BMP7 administration and telomerase inhibition are associated with precipitated increases of the cell cycle checkpoint protein p16 and tumor suppressor protein p53. Critical in tumor cell ageing and death (Krimpenfort et al., 2007; Xue et al., 2007), increased p16 and p53 activities are involved in telomere crisis responses coupling telomere deregulation with cell senescence and death (Smogorzewska and de Lange, 2002; Jacobs and de Lange, 2005). Thus, BMP7 may induce cancer cell ageing and death with a series of molecular and cellular events including telomerase inhibition, telomere shortening, and telomere-associated DNA damage response (Artandi et al., 2000; de Lange, 2002; Denchi and de Lange, 2007).

Our finding of BMP7-induced breast cancer cell apoptosis is consistent with previous findings that BMP7 induces the program cell death of myeloma cells (Ro et al., 2004) and prostate cancer cells (Miyazaki et al., 2004; Yang et al., 2005). In addition, we demonstrate the mechanisms whereby BMP7 induces cancer cell death that involve the repression of the hTERT gene and subsequent inhibition of telomerase activity and shortening of telomeres. We show that BMP7-induced cancer cell senescence and death are significantly reduced after hTERT gene is constitutively overexpressed to prevent telomerase inhibition. Consistently, silencing the hTERT gene induces breast cancer cell death without potentiating the effect of BMP7 on cancer cell death. In addition, an additive effect has been observed between BMP7 and hTERT shRNA on cell senescence and apoptosis, and that establishing telomerase activity by expressing hTERT does not completely eradicate BMP7-induced cell senescence and apoptosis. These data suggest that other mechanism(s) than hTERT gene repression are also involved in BMP7-induced breast cancer cell senescence and apoptosis.

We note for the first time that BMP7 exerts its inhibitory effect by a mechanism involving Smad3 nuclear signaling. BMP7 induces Smad3 phosphorylation, nuclear translocation and gene transcriptional activity in a time course occurring within an hour. As a repressor the hTERT gene, Smad3 expression induces telomerase inhibition (Li and Liu, 2007). When Smad3 is silenced, the repression of the hTERT gene and inhibition of telomerase activity induced by BMP7 is abolished. So, BMP7 stimulates the phosphorylation and nuclear accumulation of Smad3 in addition to that of Smad1/5/8, and that Smad3 mediates BMP7-induced telomerase inhibition, suggest that BMP7 is coupled with Smad3 signaling in regulating telomerase maintenance of telomere homeostasis. Our findings are consistent with previous findings that BMP7 is shown to induce phosphorylation of Smad2/3 in Barrett’s adenocarcinoma cells (Rees et al., 2006) and that BMP7 stimulates Smad3 target CAGA box promoter activity to a similar extent to that induced by TGF-β in a mammary epithelial cell line (Piek et al., 1999). In Barrett’s adenocarcinoma cells, Smad2/3 is phosphorylated in the presence of BMP7 and absence of TGF-β, but phosphorylation is inhibited with increasing concentrations of TGF-β (Rees et al., 2006). This TGF-β concentration-dependent inhibition of Smad2/3 phosphorylation may reflect complex cross-talks in signal transductions between BMP7 and TGF-β.

Whereas further studies are required to investigate the mechanism underlying the actions of BMP7 in stimulating Smad3 signaling in this and previous work (Piek et al., 1999; Rees et al., 2006) and inhibiting TGF-β-induced Smad3 signaling (Wang and Hirschberg, 2004b; Buijs et al., 2007a), several possibilities may exist to explain the different differential signal transduction. First, different receptors stimulated by BMP7, the potential presence of other cytokines, as well as other intracellular signaling proteins might be accountable under the specific conditions of different cell types. In an attempt to investigate a potential role of Smad1/5/8 in regulating the hTERT gene, our preliminary data did not show that Smad1/5/8 mediated BMP7 inhibition of telomerase activity and telomere maintenance (unpublished). Given that TGF-β induces a cross-talk of signaling to the Smad1/5/8 pathway in cancer and endothelial cells (Liu et al., 1998; Goumans et al., 2002), it is possible that BMP7 coupling to the Smad3 signaling pathway is involved in regulating gene expression program beyond that of the hTERT directly or indirectly via a gene transcription-dependent mechanism.

To investigate potential roles of the different type II receptors of the TGF-β family in mediating a regulatory program over the hTERT gene, we carried out dominant negative gene expressions of all four TGF-β RII receptors. Our findings that removal of the cytoplasmic domain of BMPRII receptor, but not other TGF-β RII or activin RII receptors, results in high hTERT gene promoter activity, high telomerase activity and long telomeres, demonstrating that BMPRII mediates an inhibitory signaling to the hTERT gene promoter. The data provide evidence to suggest a possible mechanism underpinning the relatively low levels of the hTERT gene promoter activity due to an inhibitory regulation mediated by BMPRII receptor in the breast cancer cells (Xu et al., 2008). Further, our findings that BMPRII receptor mutant blocks BMP7-induced repression of the hTERT gene promoter in both MCF-7 and PMC42 breast cancer cells demonstrate a BMP7- BMPRII receptor pathway in a negative regulation of the hTERT gene. Since ACTRIIB receptor mutation also inhibits BMP7-induced repression of the hTERT gene promoter albeit to less degree than that of BMPRII receptor, we cannot exclude a possibility that ACTRIIB receptor is also involved in mediating BMP7 negative regulation of telomerase activity which requires future studies. In conclusion, we demonstrate that BMP7 exerts a potent inhibitory effect on telomerase activity and telomere maintenance in human breast cancer cells, causing cancer cell ageing and death, via BMPRII receptor and Smad3 signaling to repression of the hTERT gene.

## MATERIALS AND METHODS

### Cytokines, RNAi, gene expression plasmids and antibodies

Bone Morphogenetic Protein (BMP) 2, 4, 5, 6, and 7 were from R&D systems (Minneapolis, MN, USA). Smad3 siRNA and control siRNA were from Ambion (Austin, TX, USA), c-myc and relevant control siRNAs were from Cellogenetic. Plasmid pEGFP-C1, pEGFP-C1-hTERT, and pEGFP-C1-hTERT small hairpin RNA (shRNA) were produced in this laboratory. For the hTERT shRNA expression construct, oligonucleotide (Table S1) was annealed and cloned to the EcoR1 site downstream of the U6 promoter. All plasmids were verified by DNA sequencing. For gene expressions, total RNA and proteins were extracted and processed for Western blotting and RT-PCR as described previously (Li et al., 2006; Xu et al., 2008). For subcellular distribution of Smad3, cell fractionation and immunofluorescence staining were conducted by differential centrifugations and standard immunocytochemistry respectively, as described elsewhere previously (Li et al., 2006; Xu et al., 2008). GFP or CFP fusion proteins were monitored by fluorescence microscopy and further verified by confocal microscopy as described previously (Yang et al., 2001). The primary antibodies of mouse anti-phospho-Smad3, mouse anti-c-myc, mouse anti-p53, and mouse anti-p21 were from Santa Cruz Biotechnology, CA, USA. The primary antibodies of rabbit anti-p16 were from Cell Signaling Technology, MA, USA, mouse anti-actin from Chemicon, and horseradish peroxidise-coupled second antibodies from Dako.

### Generation of TGFβ family receptor R2 dominant negative mutants

The plasmid construct encoding the first 172 amino acids of BMPRII fused to EGFP was provided by Claude Labrie (Universite Laval, Quebec, Canada). The sequences of BMPRII, ACTRIIA, ACTRIIB and TGFβRII were aligned, and constructs encoding similarly truncated proteins were produced as fusions to GFP or CFP as indicated in individual experiments. PCR was performed using HeLa cell cDNA for ACTRIIA 1-161, ACTRIIB 1-160 and TGFβRII 1-214 were performed, and the PCR products were on-gel purified, digested using EcoRI and BamHI (NEB) and cloned into the same sites in pEGFP-N1.

### Cell culture, treatment, transfection and sorting

Human breast cancer MCF-7 cell line was grown in 5% CO_2_ atmosphere at 37°C in Dulbecco’s modified Eagle’s medium (Invitrogen) containing 0.5% fetal bovine serum in 6-well plastic plates or 10-cm dishes. Cells were treated with purified recombinant cytokines at different concentrations added into culture medium in a one tenth volume for 1–4 days. Pulsatile chonic treatment was carried out by incubating cultured cells with BMP7 for 15 h followed by replacing with fresh medium and further cultures of two days. The treatment was repeated in two days continuously for two weeks. Cell transfection was conduced using Lipofectamine-2000 (Invitrogen) according to the manufacturer’s instruction. After a 24-h transfection, GFP transfected cells were sorted using FACSAria flow cytometer (BD Biosciences, San Jose, CA). The GFP positive cells were re-seeded into 6-well plates at a density of 0.2 × 10^6^ cells/mL in DMEM plus 10% FBS including Gentamicin antibiotics (Pfizer, Australia). Cells were treated with cytokines for different periods and analysed in Western blotting and semi-quantitative RT-PCR analysis to detect gene expressions as indicated in individual experiments. For subcellular distribution between cytoplasm and nucleus, cell fractionation was performed by differential centrifugation.

### Cell senescence and apoptosis analysis

β-Galactosidase staining was performed for cell senescence by incubation cells with 2 mL of staining solution (1 mg/mL X-gal, 40 mmol/L citric acid/Na phosphate buffer, pH 6.0, 5 mmol/L potassium ferrocyanide, 5 mmol/L potassium ferricyanide, 150 mmol/L NaCl, and 150 mmol/L MgCl). The stained plates were wrapped with parafilm to protect against pH changes and incubated overnight at 37°C. The cells were rinsed and stored in PBS, and analyzed by microscopy (Nikon). Apoptotic cells were analyzed a FACSCalibur flow cytometer (BD Biosciences, San Jose, CA, USA), by incubating with annexin-V-FLUOS conjugate (Roche Diagnostic, Australia) and propidium iodide (PI) (Sigma) for 15 min at room temperature. Samples were then analyzed using FL-2 and FL-3 channel respectively. An acquisition gate was set to include ~20,000 of the centrally located cells for each sample acquisition using linear forward scatter versus linear side scatter. This acquisition strategy resulted in ~40,000 ungated events being included for each sample analysis

### Luciferase gene reporter assay

The p(CAGA)13 Luc TGF-β-inducible luciferase reporter construct (0.5 µg) was transfected into cultured breast cancer cells to determine Smad3 signaling as described previously (Li et al., 2006). Briefly, cells were co-transfected with Renilla luciferase and control reporter (pRL-TK) for 48 h. During the last 24 h of 48 h transfection, cells were treated with different cytokines indicated in individual experiments. Human TERT promoter activity was measured from the recombinant hTERT promoter p330-Luc (Genebank ACCESSION AB018788) upstream of Renilla luciferase reporter gene. Transfections were carried out using LipofectAMINE2000 (DNA:LipofectAMINE2000 = 1:3) as described in the user manual. The dual-luciferase assay was analysed using Wallac Victor Light plate reader (Perkin-Elmer, United States of America).

### Telomerase activity and telomere length analysis

Telomerase activity was determined by TRAP as described previously (Li et al., 1997). For telomeres in cultured cells, metaphase spreads from cycling MCF-7 cells treated with or without BMP7 (30 ng/mL, 15 h) thee times per week for two weeks were generated using standard laboratory protocols. The slides of metaphase cells or tumor sections were fixed in 4% formaldehyde before treatment with acidified 1% pepsin solution, and hybridized with probe solution (0.3 µg/mL Cy3-conjugated [CCCTAA]_3_ PNA probe (Panagene, Daejeon, South Korea), 70% formamide, 20 mmol/L Tris-HCl, pH 7.0, 1% BSA). Washing was conducted in PBS/tween-20 with one high stringency wash at 57°C. DNA was counterstained with DAPI and visualized and captured using Nikon Eclipse TE2000 microscope, Plan Fluor 40× objective, DS-5MC CCD camera and NIS-Elements F 2.20 software (Nikon). Telomere images were captured with a Plan Fluor 100× oil-emersion objective, and individual telomere fluorescence was integrated using spot IOD analysis in the TFL-Telo 2.2 program (gift from Dr. Peter Lansdorp, Vancouver) (Rufer et al., 1998). Images from at least 13 metaphase spreads from each data point were quantified before assembly of data in a standard spreadsheet program. At least 50 nuclei from each condition were analyzed. Average length of telomere repeat fragment (TRF) from mass cell cultures were determined by in-gel hybridization following collection of total genomic DNA, HindF1/RSA1 digestions and pulse-field agarose gel electrophoresis, using radioisotope-labelled oligonucleotide probe (sequence in Table S1). The mean TRF length was assessed using the formula TRF = Σ (OD)/Σ (OD/L) as described elsewhere (Roche Diagnostic, Australia).

### Statistical analysis

Data were analysed using student t-tests for difference between two means, and Kolmogorov-Smirnov test using GraphPad Prism for difference between telomere fluorescence distributions. A probability (P) value of at least less than 0.05 was considered statistically significant.

## Electronic supplementary material

Below is the link to the electronic supplementary material.

Supplementary material 1 (PDF 8 kb)

## ABBREVIATIONS

BMPs: bone morphogenetic proteins
hTERT: human telomerase reverse transcriptase
Smads: gene products similar to the Sma genes in Caenorhabditis elegans and mothers against dpp (Mad) in Drosophila
TGF-β: transforming growth factor-β
TRAP: telomeric repeat amplification protocol

## References

[CR1] Alarmo EL, Rauta J, Kauraniemi P, Karhu R, Kuukasjarvi T, Kallioniemi A (2006). Bone morphogenetic protein 7 is widely overexpressed in primary breast cancer. Genes Chomosomes Cancer.

[CR2] Alarmo EL, Kuukasjarvi T, Karhu R, Kallioniemi A (2007). A comprehensive expression survey of bone morphogenetic proteins in breast cancer highlights the importance of BMP4 and BMP7. Breast Cancer Res Treat.

[CR3] Artandi SE, Chang S, Lee SL, Alson S, Gottlieb GJ, Chin L, DePinho RA (2000). Telomere dysfunction promotes non-reciprocal translocations and epithelial cancers in mice. Nature.

[CR4] Attisano L, Wrana JL (2002). Signal transduction by the TGF-beta superfamily. Science.

[CR5] Bestilny LJ, Brown CB, Miura Y, Robertson LD, Riabowol KT (1996). Selective inhibition of telomerase activity during terminal differentiation of immortal cell lines. Cancer Res.

[CR6] Biswas S, Guix M, Rinehart C, Dugger TC, Chytil A, Moses HL, Freeman ML, Arteaga CL (2007). Inhibition of TGF-beta with neutralizing antibodies prevents radiation-induced acceleration of metastatic cancer progression. J Clin Invest.

[CR7] Blackburn EH, Greider CW, Szostak JW (2006). Telomeres and telomerase: the path from maize, Tetrahymena and yeast to human cancer and aging. Nat Med.

[CR8] Blasco MA (2005). Telomeres and human disease: ageing, cancer and beyond. Nat Rev Genet.

[CR9] Borah S, Xi L, Zaug AJ, Powell NM, Dancik GM, Cohen SB, Costello JC, Theodorescu D, Cech TR (2015). Cancer. TERT promoter mutations and telomerase reactivation in urothelial cancer. Science.

[CR10] Buijs JT, Henriquez NV, van Overveld PG, van der Horst G, Que I, Schwaninger R, Rentsch C, Ten Dijke P, Cleton-Jansen AM, Driouch K (2007). Bone morphogenetic protein 7 in the development and treatment of bone metastases from breast cancer. Cancer Res.

[CR11] Buijs JT, Rentsch CA, van der Horst G, van Overveld PG, Wetterwald A, Schwaninger R, Henriquez NV, Ten Dijke P, Borovecki F, Markwalder R (2007). BMP7, a putative regulator of epithelial homeostasis in the human prostate, is a potent inhibitor of prostate cancer bone metastasis in vivo. Am J Pathol.

[CR12] Cheng D, Zhao Y, Wang S, Jia W, Kang J, Zhu J (2015). Human telomerase reverse transcriptase (hTERT) transcription requires Sp1/Sp3 binding to the promoter and a permissive chomatin environment. J Biol Chem.

[CR13] DamLe RN, Temburni S, Banapour T, Paul S, Mongini PK, Allen SL, Kolitz JE, Rai KR, Chiorazzi N (2012). T-cell independent, B-cell receptor-mediated induction of telomerase activity differs among IGHV mutation-based subgroups of chonic lymphocytic leukemia patients. Blood.

[CR14] de Lange T (2002). Protection of mammalian telomeres. Oncogene.

[CR15] Denchi EL, de Lange T (2007). Protection of telomeres though independent control of ATM and ATR by TRF2 and POT1. Nature.

[CR16] Dennler S, Itoh S, Vivien D, ten Dijke P, Huet S, Gauthier JM (1998). Direct binding of Smad3 and Smad4 to critical TGF beta-inducible elements in the promoter of human plasminogen activator inhibitor-type 1 gene. EMBO J.

[CR17] Galliher AJ, Schiemann WP (2007). Src phosphorylates Tyr284 in TGF-beta type II receptor and regulates TGF-beta stimulation of p38 MAPK during breast cancer cell proliferation and invasion. Cancer Res.

[CR18] Goumans MJ, Valdimarsdottir G, Itoh S, Rosendahl A, Sideras P, ten Dijke P (2002). Balancing the activation state of the endothelium via two distinct TGF-beta type I receptors. EMBO J.

[CR19] Hogan BL (1996). Bone morphogenetic proteins in development. Curr Opin Genet Dev.

[CR20] Holt SE, Wright WE, Shay JW (1996). Regulation of telomerase activity in immortal cell lines. Mol Cell Biol.

[CR21] Horn S, Figl A, Rachakonda PS, Fischer C, Sucker A, Gast A, Kadel S, Moll I, Nagore E, Hemminki K (2013). TERT promoter mutations in familial and sporadic melanoma. Science.

[CR22] Hu B, Tack DC, Liu T, Wu Z, Ullenbruch MR, Phan SH (2006). Role of Smad3 in the regulation of rat telomerase reverse transcriptase by TGFbeta. Oncogene.

[CR23] Huang FW, Hodis E, Xu MJ, Kryukov GV, Chin L, Garraway LA (2013). Highly recurrent TERT promoter mutations in human melanoma. Science.

[CR24] Jacob S, Nayak S, Kakar R, Chaudhari UK, Joshi D, Vundinti BR, Fernandes G, Barai RS, Kholkute SD, Sachdeva G (2016). A triad of telomerase, androgen receptor and early growth response 1 in prostate cancer cells. Cancer Biol Ther.

[CR25] Jacobs JJ, de Lange T (2005). p16INK4a as a second effector of the telomere damage pathway. Cell Cycle.

[CR26] James D, Levine AJ, Besser D, Hemmati-Brivanlou A (2005). TGFbeta/activin/nodal signaling is necessary for the maintenance of pluripotency in human embryonic stem cells. Development.

[CR27] Janzen V, Forkert R, Fleming HE, Saito Y, Waring MT, Dombkowski DM, Cheng T, DePinho RA, Sharpless NE, Scadden DT (2006). Stem-cell ageing modified by the cyclin-dependent kinase inhibitor p16INK4a. Nature.

[CR28] Krimpenfort P, Ijpenberg A, Song JY, van der Valk M, Nawijn M, Zevenhoven J, Berns A (2007). p15Ink4b is a critical tumour suppressor in the absence of p16Ink4a. Nature.

[CR29] Kyo S, Inoue M (2002). Complex regulatory mechanisms of telomerase activity in normal and cancer cells: how can we apply them for cancer therapy?. Oncogene.

[CR30] Kyo S, Takakura M, Taira T, Kanaya T, Itoh H, Yutsudo M, Ariga H, Inoue M (2000). Sp1 cooperates with c-Myc to activate transcription of the human telomerase reverse transcriptase gene (hTERT) [in process citation]. Nucleic Acids Res.

[CR31] Li H, Liu JP (2007). Mechanisms of action of TGF-beta in cancer: evidence for Smad3 as a repressor of the hTERT gene. Ann NY Acad Sci.

[CR32] Li H, Zhao LL, Funder JW, Liu JP (1997). Protein phosphatase 2A inhibits nuclear telomerase activity in human breast cancer cells. J Biol Chem.

[CR33] Li H, Pinto AR, Duan W, Li J, Toh BH, Liu JP (2005). Telomerase down-regulation does not mediate PC12 pheochomocytoma cell differentiation induced by NGF, but requires MAP kinase signalling. J Neurochem.

[CR34] Li H, Xu D, Li J, Berndt MC, Liu JP (2006). Transforming growth factor beta suppresses human telomerase reverse transcriptase (hTERT) by Smad3 interactions with c-Myc and the hTERT gene. J Biol Chem.

[CR35] Liu X, Yue J, Frey RS, Zhu Q, Mulder KM (1998). Transforming growth factor beta signaling though Smad1 in human breast cancer cells. Cancer Res.

[CR36] Ma T, Gutnick J, Salazar B, Larsen MD, Suenaga E, Zilber S, Huang Z, Huddleston J, Smith RL, Goodman S (2007). Modulation of allograft incorporation by continuous infusion of growth factors over a prolonged duration in vivo. Bone.

[CR37] Massague J, Seoane J, Wotton D (2005). Smad transcription factors. Genes Dev.

[CR38] Miyazaki H, Watabe T, Kitamura T, Miyazono K (2004). BMP signals inhibit proliferation and in vivo tumor growth of androgen-insensitive prostate carcinoma cells. Oncogene.

[CR39] Molofsky AV, Slutsky SG, Joseph NM, He S, Pardal R, Krishnamurthy J, Sharpless NE, Morrison SJ (2006). Increasing p16INK4a expression decreases forebrain progenitors and neurogenesis during ageing. Nature.

[CR40] Notting I, Buijs J, Mintardjo R, van der Horst G, Vukicevic S, Lowik C, Schalij-Delfos N, Keunen J, van der Pluijm G (2007). Bone morphogenetic protein 7 inhibits tumor growth of human uveal melanoma in vivo. Invest Ophthalmol Vis Sci.

[CR41] Ogawa D, Nomiyama T, Nakamachi T, Heywood EB, Stone JF, Berger JP, Law RE, Bruemmer D (2006). Activation of peroxisome proliferator-activated receptor gamma suppresses telomerase activity in vascular smooth muscle cells. Circ Res.

[CR42] Parsch D, Fellenberg J, Brummendorf TH, Eschlbeck AM, Richter W (2004). Telomere length and telomerase activity during expansion and differentiation of human mesenchymal stem cells and chondrocytes. J Mol Med.

[CR43] Patel SR, Dressler GR (2005). BMP7 signaling in renal development and disease. Trends Mol Med.

[CR44] Piek E, Moustakas A, Kurisaki A, Heldin CH, ten Dijke P (1999). TGF-(beta) type I receptor/ALK-5 and Smad proteins mediate epithelial to mesenchymal transdifferentiation in NMuMG breast epithelial cells. J Cell Sci.

[CR45] Rees JR, Onwuegbusi BA, Save VE, Alderson D, Fitzgerald RC (2006). In vivo and in vitro evidence for transforming growth factor-beta1-mediated epithelial to mesenchymal transition in esophageal adenocarcinoma. Cancer Res.

[CR46] Ro TB, Holt RU, Brenne AT, Hjorth-Hansen H, Waage A, Hjertner O, Sundan A, Borset M (2004). Bone morphogenetic protein-5, -6 and -7 inhibit growth and induce apoptosis in human myeloma cells. Oncogene.

[CR47] Rothhammer T, Wild PJ, Meyer S, Bataille F, Pauer A, Klinkhammer-Schalke M, Hein R, Hofstaedter F, Bosserhoff AK (2007). Bone morphogenetic protein 7 (BMP7) expression is a potential novel prognostic marker for recurrence in patients with primary melanoma. Cancer Biomark.

[CR48] Rufer N, Dragowska W, Thornbury G, Roosnek E, Lansdorp PM (1998). Telomere length dynamics in human lymphocyte subpopulations measured by flow cytometry. Nat Biotechnol.

[CR49] Shay JW, Wright WE (2006). Telomerase therapeutics for cancer: challenges and new directions. Nat Rev Drug Discov.

[CR50] Shi Y, Massague J (2003). Mechanisms of TGF-beta signaling from cell membrane to the nucleus. Cell.

[CR51] Simic P, Culej JB, Orlic I, Grgurevic L, Draca N, Spaventi R, Vukicevic S (2006). Systemically administered bone morphogenetic protein-6 restores bone in aged ovariectomized rats by increasing bone formation and suppressing bone resorption. J Biol Chem.

[CR52] Smogorzewska A, de Lange T (2002). Different telomere damage signaling pathways in human and mouse cells. EMBO J.

[CR53] Sugimoto H, Grahovac G, Zeisberg M, Kalluri R (2007). Renal fibrosis and glomerulosclerosis in a new mouse model of diabetic nephopathy and its regression by bone morphogenic protein-7 and advanced glycation end product inhibitors. Diabetes.

[CR54] Takakura M, Kyo S, Inoue M, Wright WE, Shay JW (2005). Function of AP-1 in transcription of the telomerase reverse transcriptase gene (TERT) in human and mouse cells. Mol Cell Biol.

[CR55] Varga AC, Wrana JL (2005). The disparate role of BMP in stem cell biology. Oncogene.

[CR56] Wang S, Hirschberg R (2004). Bone morphogenetic protein-7 signals opposing transforming growth factor beta in mesangial cells. J Biol Chem.

[CR57] Wang S, Hirschberg R (2004). Bone morphogenetic protein-7 signals opposing transforming growth factor beta in mesangial cells. J Biol Chem.

[CR58] Wu KJ, Grandori C, Amacker M, Simon-Vermot N, Polack A, Lingner J, Dalla-Favera R (1999). Direct activation of TERT transcription by c-MYC. Nat Genet.

[CR59] Xu D, Dwyer J, Li H, Duan W, Liu JP (2008). Ets2 maintains hTERT gene expression and breast cancer cell proliferation by interacting with c-Myc. J Biol Chem.

[CR60] Xue W, Zender L, Miething C, Dickins RA, Hernando E, Krizhanovsky V, Cordon-Cardo C, Lowe SW (2007). Senescence and tumour clearance is triggered by p53 restoration in murine liver carcinomas. Nature.

[CR61] Yang Z, Li H, Chai Z, Fullerton MJ, Cao Y, Toh BH, Funder JW, Liu JP (2001) Dynamin II regulates hormone secretion in neuroendocrine cells. J Biol Chem 276, 4251–4260 10.1074/jbc.M006371200 11032832

[CR62] Yang S, Zhong C, Frenkel B, Reddi AH, Roy-Burman P (2005). Diverse biological effect and Smad signaling of bone morphogenetic protein 7 in prostate tumor cells. Cancer Res.

[CR63] Zeisberg EM, Tarnavski O, Zeisberg M, Dorfman AL, McMullen JR, Gustafsson E, Chandraker A, Yuan X, Pu WT, Roberts AB (2007). Endothelial-to-mesenchymal transition contributes to cardiac fibrosis. Nat Med.

