# Supplementary material for: TGF-beta receptor mediated telomerase inhibition, telomere shortening and breast cancer cell senescence
Source: Protein Cell. 2016 Sep 30;8(1):39–54. doi: 10.1007/s13238-016-0322-1 (PMC5233610; doi:10.1007/s13238-016-0322-1)
Supplement: Supplementary file 1 — Supplementary material 1 (PDF 8 kb) [file 13238_2016_322_MOESM1_ESM.pdf]

Supplementary Table 1: Primer sequences

Gene	Aim	Primer sequence
hTERT	shRNA	5'-AATTCAAAAAGGGTCTTTCTACCAGAGGTGCTTCTCTTGAAATCATCTCTGGTAGCAAGACC-3
hTERT	PCR	5'CCACCTTGACAA-AGTACAG3' and 5'CGTCCAGACTCCGCTTCAT3
Smad3	RCR	5'CCGAATCCGATGTC-CCC3' and 5'CCCCTCCGATGTAGTAGAGCC3'
Actin	PCR	5'GCTCGTCGTCGACA-ACGGCTC3' and 5'CAAACATGATCTGGGTCSTCTTCTC3'
ActRIIA(1-161)	PCR	5-AAGAATTCAAATGGGAGCTGCTGCAAAG-3 and 5-AAGGATCCCTGTACACCCAAAATGCACAAAT G-3
ActRIIB(1-160)	PCR	5- AAGAATTCACATGACGGCGCCCTGGGTG-3 and 5-AAGGATCCCGGTACATCCAAAAGGCCAGCAG-3
TGFbRII(1-214)P16	PCR	5- AAGAATTCCCATGGGTCGGGGGCTGCTCAGG-3 and 5-AAGGATCCCGGTAGCAGTAGAAGATGATGATGAC-3
Telomere	Probe	5' CCCTAACCCTAACCCTAA 3'
CMV promoter	Sequencing	CGC AAA TGG GCG GTA GGC GTG
